# Contrast drainage through pre-existing transseptal route during left bundle branch area pacing

**DOI:** 10.1016/j.hrcr.2023.10.031

**Published:** 2023-11-02

**Authors:** Masahiro Kimura, Chisato Miura, Tomohiro Osaki, Teruki Takeda, Hiroshi Mabuchi

**Affiliations:** ∗ Department of Cardiovascular Medicine, Koto Memorial Hospital, Higashiomi, Japan; † Department of Clinical Engineering, Koto Memorial Hospital, Higashiomi, Japan

**Keywords:** Coronary vein infringement, Interventricular septal perforation, Lead dislodgement, Left bundle branch pacing, Septal injury

**Key Teaching Points**

• Though left bundle branch area pacing (LBBAP) is a highly successful technique, deep septal lead deployment sometimes causes complications that are less frequently encountered.
• The diameter of the septal tunnel by lead perforation during LBBAP would be sealed after lead retraction.
• The guiding sheath should be pulled back from the right septum to avoid complications of septal injury with venous infringement.

## Introduction

Left bundle branch area pacing (LBBAP) is an emerging method of physiological pacing that has overcome the low success rate and higher threshold issues of traditional His bundle pacing.^1^ Inducing more physiological left ventricular contractions through LBBAP could potentially reduce the risk of pacing-induced cardiomyopathy in patients with a high pacing prevalence, as compared to conventional right ventricular pacing. Although the LBBAP technique is rapidly gaining popularity worldwide, unanticipated complications related to the insertion of screw-in leads into the deep septal area have also been reported. Here we describe a case of atypical septum injury caused by contrast injection, with visualization of the route by the retracted lead from the ventricular septum.

## Case report

**Figure 1:**
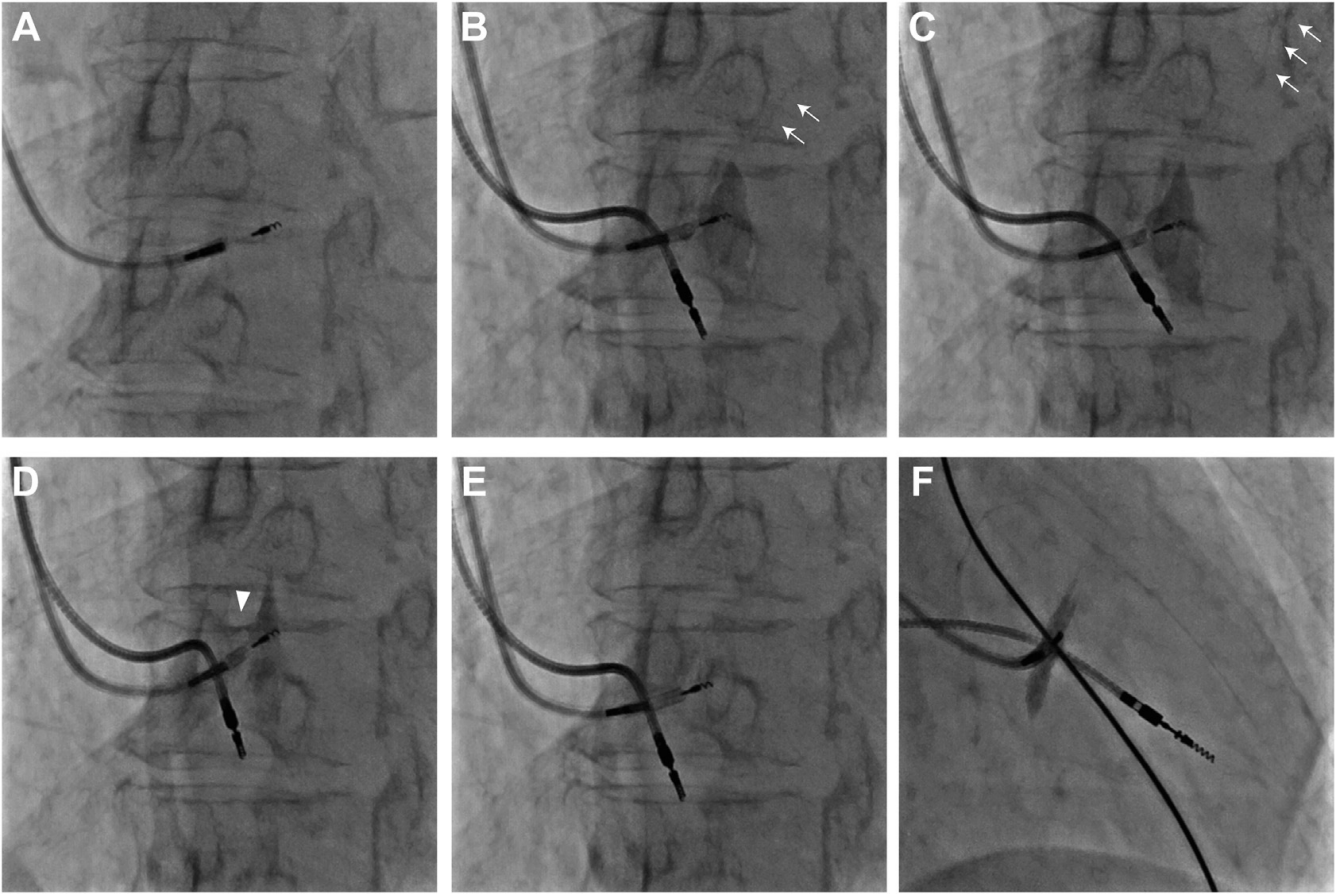
**A:** Initial lead position and the lead was retracted owing to septal perforation. **B–F:** Sequential images of contrast media injection with septal retention and immediate wash-out: left anterior oblique view **(B–E)** and right anterior oblique view **(F).** Retrograde filling of coronary veins (*white arrow*) and septal tunnel developed by the initial lead (*arrowhead*) are shown.

A 68-year-old female patient with symptoms of dizziness, palpitations, and exertional dyspnea underwent pacemaker implantation owing to complete atrioventricular block. Her echocardiography showed the left ventricular ejection fraction (LVEF) was 69%, and the septal wall thickness measured 7 mm. LBBAP was attempted using a C315HIS guiding sheath and SelectSecure 3830 lead (Medtronic Inc, Minneapolis, MN). Adequate QRS narrowing was achieved at the initial lead placement with a left ventricular activation time (LVAT) of 77 ms. However, as the lead was advanced deeper into the septum, lead impedance suddenly decreased from 660 Ω to 330 Ω, resulting in unstable pacing with ventricular capture loss even under constant pacing at 3.0 V (0.4 ms pulse width). Upon injection of contrast medium through the guiding catheter, the lead was found to be screwed into the septum at a depth of 11 mm (Figure 1A, Supplemental Video 1). Owing to the suspicion of perforation, the lead was removed.

**Figure 2:**
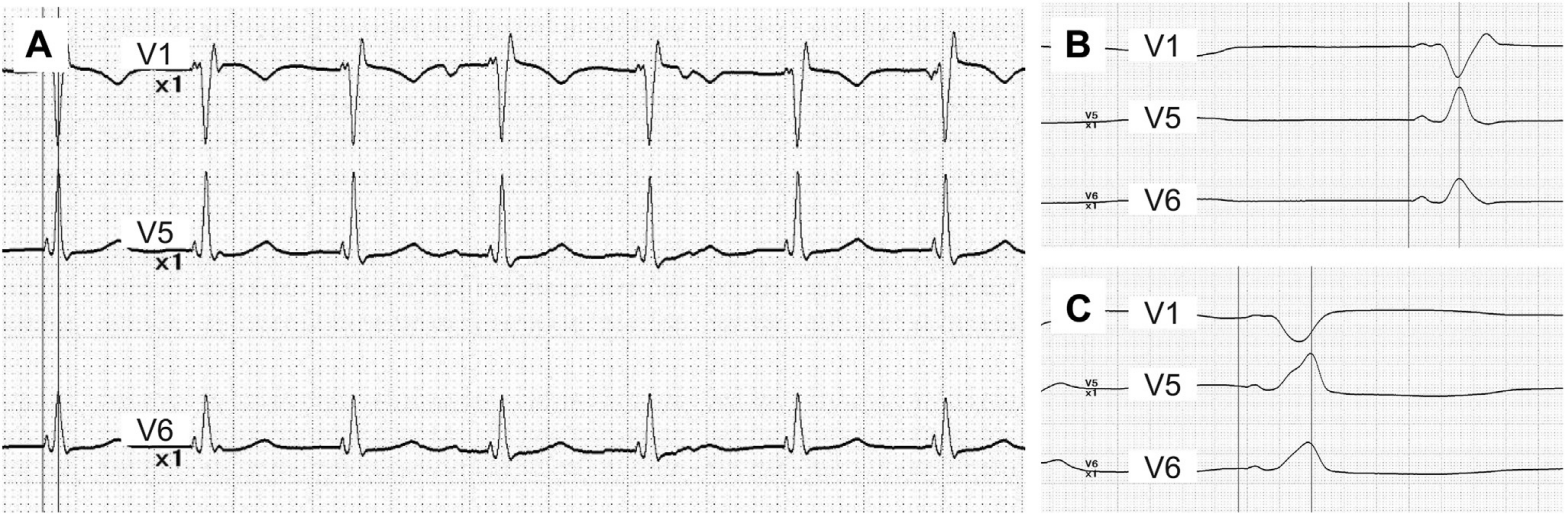
**A:** Electrocardiogram (ECG) of successful left bundle branch area pacing by repositioned lead. **B:** Typical qR pattern in V_1_ lead and with a left ventricular activation time (LVAT) of 78 ms was observed. **C:** A change of ECG morphology with prolongation of LVAT after injection of contrast material into septum.

Approximately 5 mm below the initial placement, the lead was repositioned with an LVAT of 78 ms and impedance of 570 Ω, both showing good values (Figure 2A and 2B). For the purpose of verifying the lead placement depth, contrast medium was again introduced via the guiding catheter. This revealed staining within the septal wall and coronary vein opacification, with the contrast rapidly washing out (Figure 1B–1F). Notably, the contrast was cleared out through the transseptal path formed by the initial lead insertion, flowing into the right ventricle, with no leakage observed into the left ventricle (Supplemental Video 2). Lead impedance dropped to 475 Ω, and LVAT extended to 113 ms along with the disappearance of the late R wave in the V_1_ lead (Figure 2C). As a result, the lead was extracted and repositioned at the right ventricular apical septum.

Immediate right ventriculography was performed, but no evident abnormalities were observed. No chest pain occurred, and transthoracic echocardiography did not reveal any signs of septal hematoma, perforation, or pericardial effusion; then the procedure was concluded. After the implantation, her symptoms improved, and the brain natriuretic peptide levels decreased from 136 pg/mL to 38 pg/mL, leading to her discharge at postoperative day 7. The patient was well, without any symptoms, at 1 month after implant.

## Discussion

In this report, 2 complications were encountered during the LBBAP procedure: (1) intraoperative septal perforation, and (2) acute septum injury with coronary vein visualization by contrast injection. According to previous observational studies, the prevalence of septal perforation and injury is 3.67% and 0.65%, respectively.^1^^,^^2^ Although these complications are generally considered benign, with serious adverse outcomes being rare, the pacing instability compelled us to halt LBBAP in our case.

LBBAP has gained popularity as cardiac conduction system pacing (CSP) owing to its technical attainability, stability of pacing parameters, relatively short learning curve, and potential effect to reduce the risk of pacing-induced cardiomyopathy. According to recent HRS/APHRS/LAHRS guidelines, CSP is recommended for patients who require a substantial amount of ventricular pacing with moderately reduced ventricular contraction (LVEF 36%–50%) as a Class 2a indication.^3^ Although we have somewhat limited evidence regarding the utility of LBBAP in patients with an LVEF above 50%, some observational studies have indicated the effectiveness of LBBAP for such population.^4^, ^5^, ^6^ Therefore, in cases of high prevalence of ventricular pacing, treating the patients who have preserved LVEF with CSP is deemed reasonable, as per Class 2b indication in the HRS/APHRS/LAHRS guideline.

While acute lead perforation into the left ventricle cavity is a relatively frequent complication reported in various other studies,^7^ the septal tunnel created by a lumenless lead such as SelectSecure 3830 is small and is considered to close spontaneously without detectable shunting on postprocedural echocardiography.^8^ In this case, the contrast agent injected into the septum was unexpectedly able to exit through the route by the initial lead perforation. The contrast agent flowed only into the right ventricle, but not into the left ventricle. This could be attributed to 2 reasons: firstly, the left ventricular pressure is much higher than the right ventricular pressure; and secondly, only the screw tip of the lead, not the body of it, might escape toward the left ventricular cavity. Using cine images, the measured diameter of the septal tunnel was approximately 0.55 mm, which corresponds to about 40% of the outer diameter of the SelectSecure 3830 lead (4.1F), ie, approximately 16% in terms of the cross-sectional area of the lumen. This effect is likely attributed to the myocardium’s “self-sealing” characteristics.^9^ Right after the lead traction, the myocardium would contract and envelop the perforated site. Once a lead is screwed into the septum and subsequently retracted, the septal route created by the lead can be involved in blood shunts, such as coronary artery fistula.^10^^,^^11^ To our best knowledge, our case is the first to observe this intraseptal tunnel by contrast medium, which is difficult to detect by echocardiography.

During the LBBAP procedure, contrast injection through the guiding sheath is often performed to confirm the depth of the lead into the septum. When the lead is screwed into the septum, the tip of the guiding sheath is advanced near the entry point of the lead to achieve coaxial alignment, which improves torque response. However, there is a risk of causing intraoperative septum injury if the tip of the sheath contacts the septal wall during contrast injection, and the sheath should be gently pulled back from the right septum. However, in most cases patients do not complain of any symptoms and there are no changes in the electrocardiogram; and if pacing parameters remain stable, it is considered unnecessary to alter the lead’s position. In our case, following the septum injury, the pacing capture became sporadic, necessitating the lead’s repositioning. It was likely attributed to an intraseptum space caused by the injection of contrast media, which possibly resulted in a dislodgement of the lead.

Furthermore, whether contrast injection to verify the depth of the lead is necessary or not is worth discussing, because of the following reasons. First, patients undergoing pacemaker implantation are often elderly, and there is a high prevalence of concomitant renal dysfunction. Second, the 2-dimensional view of ventriculography often inadequately captures the 3-dimensional structure of the interventricular septum. This limitation makes it challenging to accurately estimate the depth of the screwed lead into the interventricular septum. Third, unipolar pacing impedance and electrogram patterns, such as the current of injury, are more useful indicators for estimating septal perforation than ventriculography. According to the literature, it is recognized that a threshold of 450 Ω is associated with high sensitivity (100%) and specificity (96.6%) for diagnosing septal perforation.^7^ Although in our case, septal injury occurred by the second contrast injection during the procedure, the potential risk of complications may rise as the number of contrast injections increases.

There have been several case reports regarding coronary vein infringement or coronary vein fistula as complications of LBBAP,^12^^,^^13^ but another report has indicated that drainage of contrast material through coronary vein system during LBBAP represents normal physiology in the absence of fistula formation, and was observed in 2.6% of the procedures.^14^ Although venous visualization is considered usually benign, a forceful injection of contrast material should be avoided. It is speculated that variations in lead or guiding sheath diameters may impact the severity of septal injury and coronary vein fistula, but there are no documented publications on this topic. In our case, the contrast agent was pushed too hard, causing the interventricular septal dissection, connecting with the initial screwed channel, and coronary vein branches on its path were visualized.

## Conclusion

We present a case involving an uncommon septum injury resulting from forced contrast injection, along with the detection of the myocardial tunnel formed by the retracted lead, which had reduced in diameter compared to the lead’s outer size. This self-sealing phenomenon is considered important for the conservative monitoring following septal perforation during LBBAP.

## Disclosures

The authors have no conflicts of interest.

## Appendix Supplementary Data

Supplemental Video 1 Contrast injection at the initial lead position with suspected septal perforation.

Supplemental Video 2 Left anterior oblique (LAO) view of contrast injection at the replaced lead position with septal injury.
